# The Clinical Role of Heart Rate Variability Assessment in Cognitively Impaired Patients and Its Applicability in Community Care Settings: A Systematic Review of the Literature

**DOI:** 10.7759/cureus.61703

**Published:** 2024-06-05

**Authors:** Amanda Attreed, Louisa R Morand, Dimity C Pond, Joachim P Sturmberg

**Affiliations:** 1 General Medicine, Central Coast Local Health District, Gosford, AUS; 2 General Medicine, Royal Brisbane and Women’s Hospital, Brisbane, AUS; 3 General Practice, Wicking Dementia Research and Training Centre, Hobart, AUS; 4 General Practice, University of Newcastle, Newcastle, AUS

**Keywords:** wearable devices, delirium, quality-of-care, community medicine, aged care, neurocardiac axis, hrv, dementia, dementia with lewy bodies, alzheimer’s diseases

## Abstract

Heart rate variability (HRV) correlates well with a person’s overall physiological function. Clinically, HRV is successfully used in acute care to identify impending infections, but little is known about its potential in the management of chronic diseases like cognitive decline/dementia. The aim of this study was to identify the best available knowledge about HRV in cognitively impaired populations that might be applied to improve clinical practice in community settings. We conducted a systematic literature search in PubMed, Embase, and Cochrane databases published from January 2009 to August 2022. Eligible studies were selected using Covidence and each study underwent qualitative assessment using the Mixed Method Appraisal Tool. At each stage of selection, each study was reviewed independently by two members of the team, and any disputes were discussed along the way. The literature identified that the brain regions controlling HRV are also those affected by dementias of Alzheimer's type (AD) and Lewy body types (DLB). HRV was impaired in both types, with DLB showing greater impairment in all HRV parameters compared to AD. No studies explored the temporal changes of HRV or its use in the clinical management of people with cognitive impairment (CI). The current lack of standardization of HRV recording and analysis limits its use in clinical practice. HRV may emerge as a potentially useful tool to identify people with early/preclinical memory impairment and help to differentiate AD from DLB. Longitudinal HRV measurement is emerging as a useful way to monitor disease progression and treatment response, and continuous HRV measurement may prove useful in the early identification of sepsis and its complications in patients no longer able to communicate their illness experiences.

## Introduction and background

Dementia is the progressive loss of cognitive function, affecting various domains of cognition and leading to significant impairment of activities of daily living [1,2]. It is an overarching diagnosis that encompasses numerous underlying pathological processes. Clinically these various forms are not always easily distinguishable, and some studies demonstrated that heart rate variability (HRV) might be a reliable biomarker to differentiate subtypes [3-5].

While decreased HRV is normal with aging [2,5-11], abnormal patterns are seen in many common diseases [12], though it has specific diagnostic patterns in cognitive decline [10,13]. Of pragmatic importance is the use of HRV in the early detection of impending sepsis [14-16] which is associated with a high burden of morbidity and a marked increased risk of otherwise avoidable mortality [17].

Despite the usefulness of HRV measurement in acute care, it has not yet been widely adopted in other settings including aged care. This raises the question: Could HRV measurement and monitoring play a role in the care of patients with cognitive impairment (CI) and dementia?

Central control of HRV

The study of systems biology, a biology-based interdisciplinary field that investigates the complex interactions of multi-level biological systems including intracellular, intercellular, hormonal, and macroscopic interactions has established HRV as a valuable tool in demonstrating such relationships [10,18-20]. A healthy individual demonstrates considerable HRV which declines in periods of illness [21]. However, almost any disease [11,12], irrespective of cardiac involvement [22] is associated with a persistent decline in HRV, and there is a marked decline in HRV with aging [6,7,9] (loss of complexity hypothesis of aging [6]). Superimposed on this is the loss of variability seen in illness [12]).

**Figure 1:**
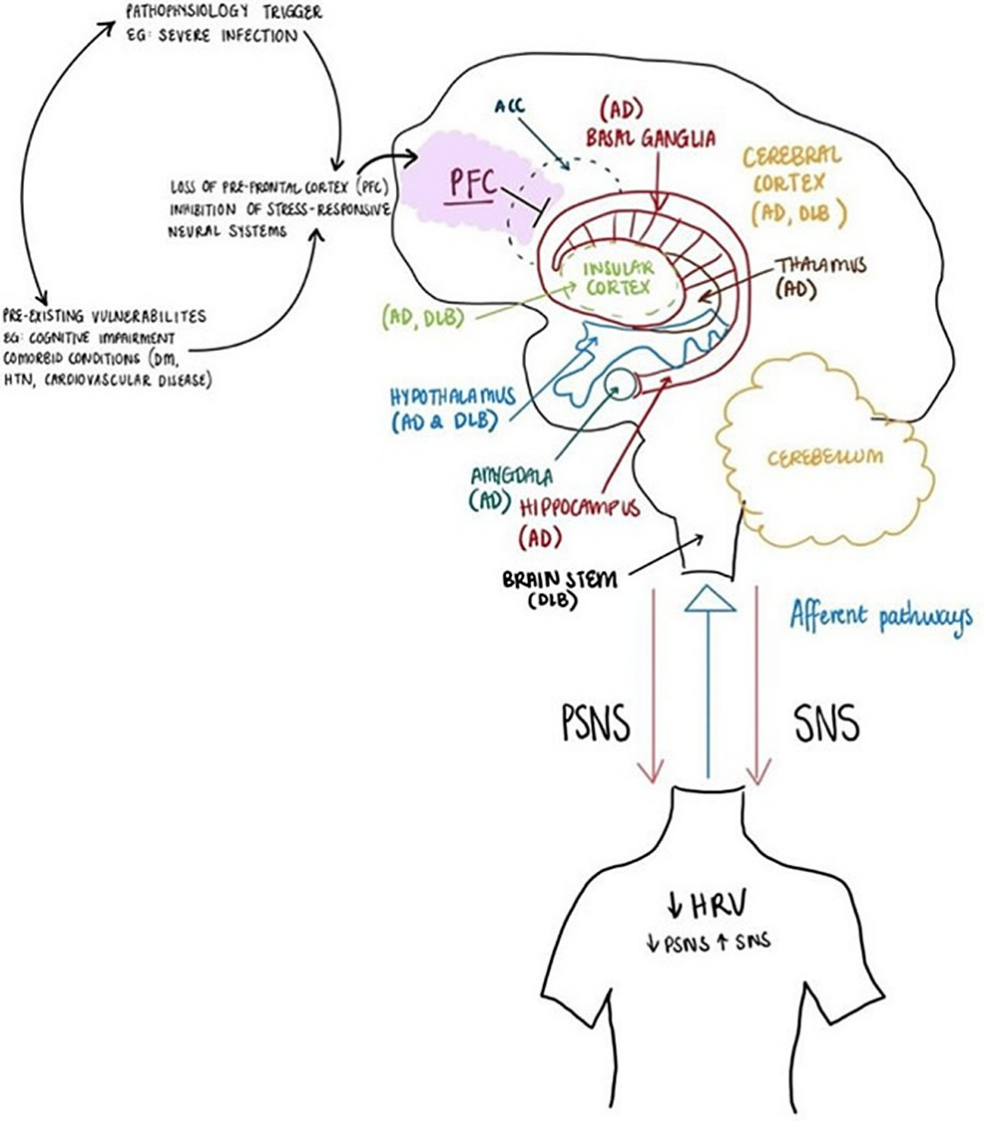
CNS control of HRV showing the overlap between the neurocardiac axis and brain regions impacted by cognitive impairment (CI) AZ: Alzheimer's disease; ACC: anterior cingulate cortex; DLB: dementia with Lewy bodies; HRV: heart rate variability; PFC: prefrontal cortex; PSNS: parasympathetic nervous system; SNS: sympathetic nervous system Credit: Image created by authors

The neurocardiac axis consists of the prefrontal cortex, insular cortex, anterior cingulate cortex, and amygdala, which play a vital role in autonomic function and thus HRV (Figure 1) [2,23-26]. In particular, insular cortex damage or dysfunction is associated with increased sympathetic and decreased parasympathetic tone [24,25] whereas prefrontal cortex damage/dysfunction leads to increased high frequency-HRV (HF-HRV) due to the removal of the parasympathetic nervous system (PNS) innervation [27].

HRV in cognitive impairment

HRV mirrors the autonomic balance between sympathetic and parasympathetic pressures on the heart [19], as measured through analysis of fluctuating inter-beat intervals [28]. It is increasingly viewed as an indicator of an individual's overall physiological state, with greater variability a sign of better physiological health [4,29].

Dementia affects all parts of the brain including those neuroanatomical structures that control HRV and explains the established relationship between decreased autonomic and cognitive function [24,30]. However, it remains unclear whether this reflects a causal relationship as postulated by the hypothesis of cerebral hypoperfusion [2,31,32], or simply whether both result from separate underlying processes. While the pathological aggregation of *plagues *(amyloid) and *tangles* (tau proteins) is associated with AD, Lewy body proliferation with dementia with Lewy bodies (alpha-synuclein) (DLB), vascular damage with vascular dementia (VD), and neuroinflammation emerging as an important co-contributing pathway [33], all-cause reduction in overall neuronal function and a decreased capacity to maintain autonomic system complexity.

As autonomic function is decreased in people with cognitive impairment (CI) it is worth exploring whether HRV could also be an indicator of the extent of cognitive dysfunction. There is an emerging appreciation of the relationship between HRV and decreased cognitive function in the current literature, demonstrating a pattern of higher sympathetic and lower parasympathetic function in people with decreasing cognitive testing scores [11,23,34,35] as well as established dementias of Alzheimer's type (AD) [2]. These reviews all concluded that further research is needed to better understand the specific intricacies of this observed positive relationship. This systematic review, rather than re-asserting that this relationship exists, investigates whether there are any potential clinical uses for HRV measurement in cognitively impaired people.

While evidence indicates that feedback pathways between all physiological systems can result in the modulation of autonomic function [4,29], we want to examine the clinical value of measuring HRV in patients with mild cognitive impairment (MCI) and dementia. A systematic review of the literature was conducted to ascertain if HRV can contribute to an accurate diagnosis and if continuous HRV monitoring can detect early delirium when patients are no longer able to communicate their changing health experiences.

## Review

Method

Search Strategy

A search of the PubMed, Embase, and Cochrane databases, using the search terms ("Heart rate variability" OR "HRV") AND ("Cognitive impairment" OR "dementia" OR "cognitive function") for studies published from January 2009 to August 2022.

Inclusion Criteria

The inclusion criteria were studies published in English; included at least one cohort of participants considered to have MCI and/or dementia; included a comparison between different levels of cognitive functioning, either between cohorts or within a spectrum of one cohort; and directly investigated autonomic function - measured directly through HRV - and MCI and/or dementia and/or AD and/or DLB and or VD.

Exclusion Criteria

Studies excluded were those that involved an active intervention between cohorts; review studies; and studies where HRV or cognitive function was not considered the primary outcome.

Study Selection

**Figure 2:**
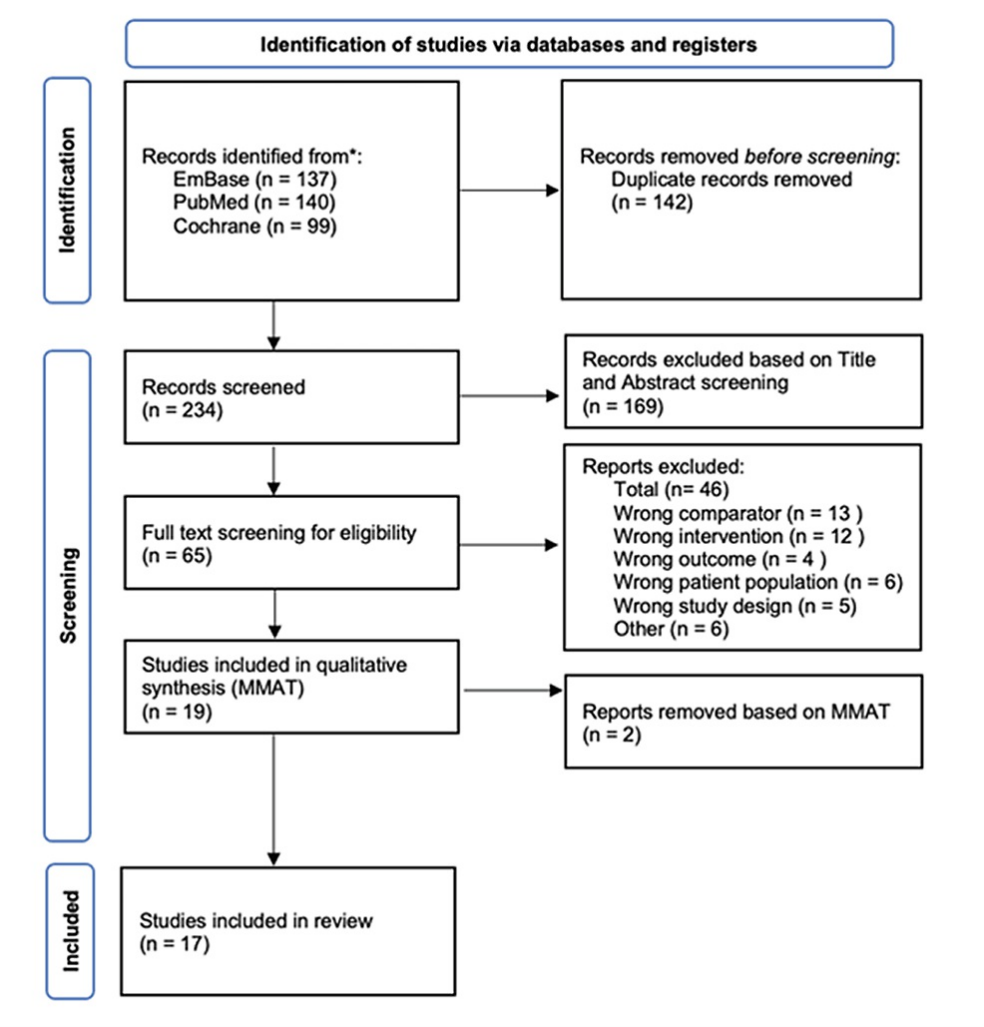
PRISMA flow chart depicting the literature search and study selection PRISMA: Preferred Reporting Items for Systematic Reviews and Meta-Analyses

A total of 17 studies were identified for this systematic review (Figure 2). Eligible studies were selected using Covidence [36] and underwent qualitative assessment using the Mixed Method Appraisal Tool (MMAT) [37]. At each stage of selection, each study was reviewed independently by two members of the team, with any disputes being discussed along the way.

CI/dementia typically occurs in a multimorbidity context where these diseases and their treatment can impact HRV. As we were interested in the "real world" applicability of HRV to better manage these patients, studies that reported comorbidities and the use of medications known to alter HRV, like acetylcholinesterase inhibitors and beta blockers, were included.

Quality assessment

We assessed all selected studies using the MMAT [36], with only two studies considered to be of inadequate quality. The MMAT assesses the methodological quality of different study designs, including assessing the validity of the research question, the method of participant collection and data collection, as well as quality of the data to appropriately address the proposed question without bias. The two studies were excluded on the basis of inadequate data collection methods and insufficient data to allow for reasonable discussion.

Results

We identified 13 cross-sectional studies [2,3,5,10,11,13,24,38-42], 3 cohort studies [8,9,32], and 1 case-control study [4] (Table 1). One of the cohort studies used the raw data gathered as part of a randomized control trial [32]. Six studies [8,9,32,39,41,42] analyzed data from previous large prospective cohort studies, and 11 gathered primary data [2,3,5,10,11,13,24,38,40,43]. Grouping of cohorts was varied depending on the study objective. Eight studies grouped participants by cognitive status e.g., controls vs. types of CI (MCI/non-amnesic mild CI (naMCI) and amnesic mild CI (aMCI) [3,4,11,13,24,38,40,43]), four studies by HRV parameters e.g., quartiles of RMSSD [8,32,41,42], and four had no grouping [2,9,10,39]. The remaining study grouped participants based on the presence or absence of white matter lesions on MRI - the extent of which has been found to be independently associated with decreased HRV in both time and frequency domains and is purported to be implicated in the pathogenesis of dementia and HRV decline [5,13,42].

**Table 1:** Summary of studies Cognitive Domains Tested: GC: global cognition; ME: memory; LG: language; A/PS: attention/processing speed; EF: executive function; VS: visuospatial skills; MMSE: Mini-Mental-State Exam; MoCA: Montreal Cognitive Assessment; RBANS: Repeatable Battery for Assessment of Neurological Status; CASI: Cognitive Abilities Screening Instrument; DSM-IV: Diagnostic and Statistical Manual of Mental Disorders 4th Edition; CAMCOG: Cambridge Cognitive Examination; EXIT-25: Exit Interview 25; DCT: Digit Coding Test; PR: Prose Recall; RCFT-DR: Rey-Osterrieth Complex Figure - Delayed Recall; VLT: Verbal Learning Test; DS-F: Digit Span - Forwards; DS-B: Digit Span - Backwards; TM-A/B: Trail Making Tests A & B; WCFST: Weigl’s Colour-form Sorting Test; CE: Cognitive Estimates; RCPM: Raven's Coloured Progressive Matrices; LF: Letter Fluency; CF: Category Fluency; PN: Picture Naming; TT: Token Test; K-BNT: Boston Naming Test; COWAT: Controlled Oral Word Association Test; BT: Bell Test; STR: Stroop Test; RCFT-C: Rey-Osterrieth Complex Figure - Copy; CGF: Copy of Geometric Figures; DRT: De Renzi’s Test; DSST: Digit Symbol Substitution Test; WMST: Wechsler Memory Scale Test; CDT: Clock Drawing Test; EXAMINER: Examiner Package; HRV Domains: SDNN: standard deviation of NN intervals; RMSSD: root mean square of successive RR interval differences; pNN50: proportion of successive RR intervals that differ by more than 50 ms; SDRR: standard deviation of RR intervals; SDANN: standard deviation of average NN intervals; SDNNI: mean of the standard deviations of NN intervals for each 5-minute segment; VLF: very low frequency; LF: low frequency; HF: high frequency; LF/HF: ratio of LF-to-HF power; TP: total power

Study	Cognitive tests used	Cognitive domains tested						HRV domains	Relationship elicited	
		GC	ME	LG	A/PS	EF	VS		Significant	Nil significance/not found
Cross-sectional studies										
Nonogaki et al., 2017 [2]	Attention: DCT & BT memory: PR, RCFT-DR, DS-F executive functions: Digit DS-B, TM-A/B, WCFST, CE, RCPM, LF language: CF, PN, TT visuospatial skills: RCFT-C, CGF ideomotor praxis: DR	+	+	+	+	+	-	LF HF LF/HF	Positive LF/HF with MMSE memory negatively with SNS (HF, LF/HF) global cognition negatively with SNS (HF, LF/HF)	HRV & executive function
Kasanuki et al., 2015 [3]	MMSE	+	-	-	-	-	-	SDNN RMSSD PNN50 LF HF LF/HF TP	DLB: decreased RMSSD, pNN50, SDNN, VLF, LF, TP Cf AD & controls reduction in HF Cf AD	n/a
Galluzzi et al., 2009 [5]	MMSE	+	-	-	-	-	-	SDANN SDRR SDNNI RMSSD LF HF LF/HF	Decreased RMSSD & LF in those with WMLs Decreased RMSSD, LF & HF with increased extent of WMLs	n/a
Dalise et al., 2020 [10]	MMSE MoCA	+	-	-	-	-	-	SDNN SDANN RMSSD LF HF LF/HF HR	Positive SDNN, SDANN, LF, LF/HF with MoCA/MMSE scores	No relationship between RMSSD or HF found
Collins et al., 2012 [11]	MMSE RBANS EXIT-25 CAMCOG	+	+	-	-	-	-	SDNN RMSSD pNN50 LF HF VLF	Positive HF in MCI Cf controls positive parasympathetic reflex test scores and executive function	Non-significant decreases in RMSSD, LF, Log LF/HF & pNN50
Nicolini et al., 2014 [13]	Attention: DCT & BT memory: PR, RCFT-DR, DS-F executive functions: Digit DS-B, TM-A/B, WCFST, CE, RCPM, LF language: CF, PN, TT visuospatial skills: RCFT-C, CGF ideomotor praxis: DR	+	+	+	+	+	+	LF HF LF/HF TP VLF LFnu HFnu	Negative HF with cognitive scores decrease in LF & LF/HF shift toward parasympathetic modulation	HRV indices at baseline
Omoya et al., 2021 [38]	MMSE	+	-	-	-	-	-	LF HF LF/HF	Reduced LF among all groups LF lower in DLB Cf AD Both SNS & PSN impaired in DLB	HF or LF/HF among all groups
Schaich et al., 2020 [39]	CASI DCT DS-F, DS-B	+	+	-	-	+	-	SDNN RMSSD	Positive SDNN with CASI positive SDNN & RMSSD with DSC score positive SDNN with DS score	Nil longitudinal change in HRV with any cognitive test score
Lin et al., 2017 [40]	MoCA STR	+	+	-	+	-	-	HF	Negative HF with cognitive performance, and increased decline in HF	Nil diff in HF at rest, however reduction during tasks in both groups
Hämmerle et al., 2021 [41]	MoCA	+	-	-	-	-	-	SDNN RMSSD HTI LF HF MHR	Positive HRVI with MoCA in both SR & AF groups	RMSSD, SDNN, TP, VLF, LF & HF not associated with lower cognitive function in either group
Zeki Al Hazzouri et al., 2014 [42]	MMSE VLT	+	+	-	-	-	-	MCR/R-Bar	Positive quartiles of R bar with MMSE scores	n/a
Kong et al., 2021 [43]	MMSE	+	+	-	-	-	-	RMSSD LF HF LF/HF	Positive HF during REM but not NREM or wake with cognitive scores Decreased HF and increased LF during NREM in memory-impaired MCI	HF during wake across all groups
Nicolini et al., 2020 [44]	Attention: DCT & BT memory: PR, RCFT-DR, DS-F executive functions: Digit DS-B, TM-A/B, WCFST, CE, RCPM, LF language: CF, PN, TT visuospatial skills: RCFT-C, CGF ideomotor praxis: DR	+	+	+	+	+	+	LF HF LF/HF TP VLF LFnu HFnu LF HF LF/HF	aMCI: decreased LFn, LF/HF & increased HF Cf naMCI & Control during active standing LFn & LF/HF positive correlation with Prose-delayed recall & negative correlation with DCT and Letter Fluency	HRV indices at baseline no difference found between naMCI & control in response to orthostatic challenge
Cohort studies										
Frewen et al., 2013 [8]	MoCA	+	+	+	-	+	-	SDNN LF HF LF/HF	Positive HF, SDNN, LF & LF/HF with MoCA scores, mainly in memory and language domain	n/a
Weinstein et al., 2021 [9]	DSM-IV use of National Institute guidelines on diagnosis of AD or possible AD	+	-	-	-	-	-	SDNN RMSSD	Decreased SDNN & RMSSD with dementia risk >60 years old	n/a
Mahinrad et al., 2016 [32]	MMSE STR DCT PWLT	+	+	-	+	+	-	SDNN	Positive SDNN with Stroop & Letter-Digit Coding test scores Those in lowest third of SDNN had more pronounced drop at follow-up exam after 3.2 years	HF at rest
Cohort studies										
Kim et al., 2018 [4]	SVLT RCFT COWAT K-BNT STR	+	+	+	+	+	+	SDNN RMSSD LF HF LF/HF	Overall time & frequency reduced in DLB positive verbal learning test with HRV positive RMSSD, HF and TP with stroop tests scores positive COWAT-market scores with RMSSD & SDNN positive TP and LF with RCFT score in both DLB & AD	n/a

The characteristics of the study populations are consistent with those observed in clinical settings (Table 2). HRV was measured using a variety of different measures (Table 3) and techniques.

**Table 2:** Sample characteristics

Variables	Details	References
Sample size	Total across all studies: 15,841	-
	Range: 38 [40] to 4,763 [8]	-
Patient age (mean)	Mean age in study sample: 73 years	-
	Range: 61.7 [41] to 79.1 years of age [3]	-
Females	Varied, lowest 28.1% [41]	Predominately female participants [2,4,5,8,9,13,24,38-40,42,43]
Educational level	Collected in 13 studies	[2,4,5,8,9,13,24,38-40,42,43]
Clinical data	Medications	[2-5,8-11,13,24,32,39-43]
	Comorbidities	[2-5,8-11,13,24,32,39-43]
	Alcohol consumption	[8,11,13,24,41,43]
	Smoking	[4,8-11,13,24,32,39,41,43]
	Body mass index	[8-11,13,24,32,39,41-43]

**Table 3:** HRV changes with different memory loss impairments aMCI: amnestic mild cognitive impairment; AD: Alzheimer’s disease; HRV: heart rate variability; SDNN: standard deviation of NN intervals; HF: high frequency; LF: low frequency; LF/HF: ratio of LF-to-HF power; RMSSD: root mean square of successive RR interval differences; TP: total power; DLB: dementia with Lewy bodies; EF: executive function; MCI: mild cognitive impairment; naMCI: non-amnestic mild cognitive impairment

Cognitive impairment	HRV changes	Explanation
Memory impairment aMCI [11,24,43] or AD [2,4]	Greater memory impairment showed greater HRV and autonomic dysfunction	Attributed to the location of neurodegenerative changes in AD and aMCI, thought to cause both memory and central autonomic dysfunction [2,4,8,11,24,39,43]
Language	Lower SDNN, HF, LF and LF/HF was found in one [8], and Only LF/HF compared in the second [2]	Association was attributed to the early language deficits seen in aMCI [24]
Attention/processing speed [4,39]	Reduced HRV RMSSD, TP, and HF	Used to distinguish DLB cohorts from AD, and controls
Executive function [9,11,13,24,32,39]	HRV dysfunction was associated with EF impairment - particularly in studies which separated subgroups of MCI (i.e., DLB/AD, naMCI/aMCI) [4,24]	Mechanism still under investigation
Visuospatial skills [4,13,24]	associated with TP and LF in both AD and DLB cohorts	No clear explanation for observation that reduced HRV is associated with verbal memory impairment, but not visual memory [24]

Relationship between domains of memory impairment and HRV

The various subtypes of dementia, though somewhat distinguishable clinically in their later stages, may show only subtle traits early in their disease progression making management with preventative measures, or through targeted disease-slowing intervention, difficult and less effective. AD, whilst strongly associated with more global impairment and a gradual onset, may be difficult to distinguish from VD which shows less memory impairment but greater fluctuations in mood as well as physical frailty, and typically has a stepwise onset. Similarly, the visual hallucinations, Parkinsonism, and marked fluctuations in cognition that characterize DLB appear later in the disease progress. Frontotemporal dementia (FTD) demonstrates the loss of prefrontal cortex inhibition, resulting in personality changes, mood changes, apathy, and disinhibition, but also shares features of dysphasia and executive impairment common in AD [45].

DBL compared to AD revealed significantly lower HRV markers (SDNN, RMSSD, pNN50, VLF, LF, TP, and LF-HRV), which may be helpful to distinguish the two types of dementia [3,4,37], and might allow an earlier diagnosis and potentially improved patient outcome. However, no studies examined or demonstrated a relationship with any other distinguishing clinical features of the dementia subtypes.

Importantly, no study examined the prognostic value of HRV in detecting the impending development of sepsis [16], a very common and potentially life-threatening condition amongst people with CI, often resulting in severely distressing delirium and increased mortality [20].

Discussion

HRV has been postulated to be an "integrating" biomarker of overall physiological function and thus a tool to diagnose and monitor the long-term progression of health and disease [29]. In the context of CI HRV has identified characteristic patterns between CI and healthy controls [9,11] and it also shows statistically significant differences between dementia subtypes [3,4,8]. These findings indicate that HRV might emerge as a useful biomarker to diagnose, manage, and monitor patients with CI in community settings, and is supported by a recent longitudinal study by Nicolini’s research group [26].

HRV and MCI Are Tightly Linked

The neurocardiac axis, responsible for autonomic nervous system (ANS) control, overlaps anatomically with regions of the brain - the prefrontal cortex, insular cortex, and hypothalamus - implicated in both DBL and AD (see Figure 1). These regions closely communicate with the hippocampus - the region of the brain responsible for memory, and memory impairment, which is a key trait of AD [5,9,13,43,44]. Similarly, infiltration and deposition of Lewy bodies - a key component of the pathophysiology of DBL - also overlap with the neuro-cardiac axis. The damage/dysfunction of these closely interrelated brain structures has been postulated to cause a decrease in PNS innervation [46].

The underlying correlation between CI and HRV is attributed to neurodegeneration and autonomic dysfunction. However, neurodegeneration is just one component of dementia, with abnormal protein deposition also implicated [8,9,34]. However, their "causative role" in CI is contested as 20-30% of patients with identified plaques and tangles do not exhibit cognitive changes [46,47]; an alternative etiology is brain hypoperfusion [31,39,48] and neuroinflammation [33] leading to decreased brain synaptic/metabolic activity [46].

Is HRV a Tool to Differentiate DLB From AD?

There is emerging evidence that HRV can distinguish DLB and AD in the early stages of cognitive decline [3,4,8]. However, patterns are heterogeneous; DLB shows a greater decrease in almost all HRV measures with SNS more significantly affected than PNS [4,43], and a greater HRV decrease in both PNS and overall cardiac function markers [35]. While promising these findings cannot yet be reliably applied clinically, and the confirmation of DBL currently still relies on metaiodobenzylguanidine (I-MIBG scintigraphy) [4,49,50] or microneurography [43] imaging.

Practical Difficulties and Future Potentials

HRV is calculated by observing inter-beat intervals of the heart and is currently derived from 12-lead electrocardiograms. However, the rapid advances in wearable technologies allow a much broader approach to HRV monitoring.

At this stage, more research is needed before one can confidently implement HRV into routine care for CI/dementia patients. The development of a standardized HRV testing protocol is a priority - how to consider the impacts of personal characteristics (e.g., BMI), habits (smoking, alcohol consumption), co-morbidities and medications; recording length; and the role and benefits of additional provocation testing. Of equal importance is the exploration of how co-morbidities alter HRV patterns in dementing illnesses.

So far studies have only used single recordings in an individual patient which limits the diagnostic and prognostic value given the great intrapersonal variability shown on repeat recording. The impact of co-morbidities on HRV patterns requires further attention. Future studies should employ repeat or continuous HRV monitoring of at-risk populations to evaluate the validity of altered HRV patterns for the early diagnosis of CI [3,4,40,43] and the early differentiation between clinical subtypes [3,9,40].

The proven benefits of continuous HRV monitoring in predicting impending sepsis [16] are of particular clinical interest for patients with advanced CI no longer able to communicate unwellness as infections often trigger delirium [6]. The rapidly emerging technologies of wearable devices and automated HRV interpretation software will make this feasible for community practice in the not-too-distant future.

Study Limitations

This systematic review looked to identify the potential clinical uses of HRV in cognitively impaired people through extrapolating data explored in current research. While systematic reviews generally seek to minimize the variability amongst studies, this review accepted the methodological limitations of the emerging field of HRV measurement to focus on the clinical potential to improve patient care and health outcomes. As such, while our results are difficult to generalize, they nevertheless offer important pointers for future research focused on maintaining and potentially improving the quality of life of people living with CI.

HRV research in CI so far has overwhelmingly used a cross-sectional and cohort design. While these studies have demonstrated a correlation between the two, there is significant variability between patients. As it is well known that HRV decreases in the presence of almost all diseases its usefulness as a diagnostic and prognostic tool more likely arises from following individual patient’s clinical and HRV trajectories over time.

The future potential of HRV monitoring in CI in primary care settings

At this stage Forte’s interpretation of this literature that “despite these limits (to specific links of HRV to cognitive functional domains), HRV could be considered a promising early biomarker of cognitive impairment in populations without dementia or stroke” appears overly optimistic from a clinician’s perspective [33]. The literature on HRV and CI has been conducted on populations with already established diseases without clear indicators of which HRV parameters correlate with the early onset of a dementing illness.

However, there are two as yet unexplored potentials that deserve further bedside-driven attention. First, could longitudinal HRV recordings - best including autonomic nervous system (ANS) challenge tests - provide indications of the rate of cognitive decline and thus offer guidance for early advanced care planning? A 10-week-long longitudinal pilot study for the first time demonstrated the feasibility of wearables to monitor cognitive function based on HRV parameters, which may help community clinicians assess disease progression as well as treatment responses [51]. Second, given the rapidly emerging technologies of wearable devices and automated HRV-interpretation software, the emergence of infections/sepsis - especially for those no longer able to communicate deterioration in their well-being - may well be pre-emptively identified and thereby avert unnecessary morbidity and potentially premature mortality.

## Conclusions

HRV assessment in dementing illnesses is an emerging research field. Available evidence indicates its potential to identify people with early/preclinical memory impairment and to help in differentiating AD from DLB. Longitudinal HRV measurement is emerging as a useful way to monitor disease progression and treatment response. Based on the experiences in acute care settings, continuous HRV measurement may prove useful in the early identification of sepsis and its complications in patients no longer able to communicate their illness experiences.
